# Use of shared care and routine tests in follow-up after treatment for localised cutaneous melanoma

**DOI:** 10.1186/s12913-018-3291-7

**Published:** 2018-06-20

**Authors:** Wei-Yin Lim, Robin M. Turner, Rachael L. Morton, Marisa C. Jenkins, Les Irwig, Angela C. Webster, Mbathio Dieng, Robyn P. M. Saw, Pascale Guitera, Donald Low, Cynthia Low, Katy J. L. Bell

**Affiliations:** 1 Clinical Research Centre Perak, Ministry of Health Malaysia, Ipoh, Perak Malaysia; 2 School of Public Health, The University of Sydney, Sydney, NSW Australia; 3 Dunedin School of Medicine, University of Otago, Dunedin, New Zealand; 4 NHMRC Clinical Trials Centre, The University of Sydney, Sydney, NSW Australia; 5 Melanoma Institute Australia, Sydney, NSW Australia; 6 Discipline of Surgery, The University of Sydney, Sydney, NSW Australia; 7 Division of Surgery, Royal Prince Alfred Hospital, Camperdown, NSW Australia; 8 Discipline of Dermatology, The University of Sydney, Sydney, NSW Australia; 9 The Sydney Melanoma Diagnostic Centre, Royal Prince Alfred Hospital, Camperdown, NSW Australia; 10 Cancer Voices NSW, Sydney, NSW Australia; 11 Centre for Evidence Based Practice, Bond University, Gold Coast, QLD Australia; 12 The University of Sydney, Rm 333 Edward Ford Building (A27), Sydney, NSW 2006 Australia

**Keywords:** Melanoma, Follow-up studies, Diagnostic imaging, Practice patterns, general practitioners, Interdisciplinary communication, Surveys and questionnaires

## Abstract

**Background:**

Patients may decide to undertake shared care with a general practitioner (GP) during follow-up after treatment for localised melanoma. Routine imaging tests for surveillance may be commonly used despite no evidence of clinical utility. This study describes the frequency of shared care and routine tests during follow-up after treatment for localised melanoma.

**Methods:**

We randomly sampled 351 people with localised melanoma [American Joint Cancer Committee (AJCC) substages 0 - II] who had not had recurrent or new primary melanoma diagnosed from a total of 902 people diagnosed and treated for localised melanoma at a specialist centre in 2014. We interviewed participants by telephone about their experience of follow-up in the past year, and documented the proportion of patients who were undertaking shared care follow-up with a GP. We also recorded the frequency and type of investigations during follow-up. We calculated weighted estimates that are representative of the full inception cohort.

**Results:**

Of the 351 people who were invited to participate, 230 (66%) people consented to the telephone interview. The majority undertook shared care follow-up with a GP (61%). People who choose to have shared care follow-up with a GP are more likely to be male (*p* = 0.006), have lower AJCC stage (p for trend = 0.02), reside in more remote areas (p for trend< 0.001), and are less likely to have completed secondary school (*p* < 0.001). Few people saw a non-doctor health practitioner as part of their follow-up (9%). Many people report undergoing tests for melanoma, much of which may be routine tests for surveillance (37%).

**Conclusions:**

The majority of people treated for a first primary localised melanoma at a specialist centre, without recurrent or new melanoma, choose to undertake shared care follow-up with a GP. Many appear to have routine diagnostic imaging as part of their melanoma surveillance.

**Electronic supplementary material:**

The online version of this article (10.1186/s12913-018-3291-7) contains supplementary material, which is available to authorized users.

## Background

The incidence of melanoma has been increasing worldwide, largely driven by an increased detection of localised disease [American Joint Cancer Committee (AJCC) stages 0, I or II], in particular in-situ and thin invasive melanomas (Breslow thickness < 1 mm) [1–3]. This has resulted in a large and increasing number of people undergoing frequent scheduled follow-up with specialist clinicians, which may place an unnecessary burden on patients, specialists, and the healthcare system.

Less frequent scheduled visits with a specialist may be safe and cost-effective, [4–7] and can be achieved by sharing follow-up care with general practitioners (GPs) [8]. Shared follow-up has been successfully trialled in other cancers, [9] as well as melanoma [10]. Some specialist clinicians at Melanoma Institute Australia (MIA), a large Australian melanoma specialist treatment centre, already use this model of follow-up care [11, 12]. Current Australasian guidelines do not make a recommendation on the use of shared care follow-up, but recommend against routine imaging in this low risk group of melanoma survivors [13].

We aimed to investigate these issues further with the following research questions in mind: How many people treated for first primary localised melanoma at a specialist centre had shared care follow-up with a local GP, and how frequently were routine investigations used? We determined the proportion of people initially treated for localised melanoma at MIA without a diagnosis of recurrent or second primary melanoma, who had some of their follow-up outside of this centre, and how often this included shared care with a GP. We also documented the type and frequency of routine investigations used in follow-up. We hope that findings from this study will offer ideas on the coordination and delivery of follow-up care across different settings, in order to address the needs and improve the outcomes for the growing number of people treated for localised melanoma.

## Methods

### Study population and setting

**Fig. 1:**
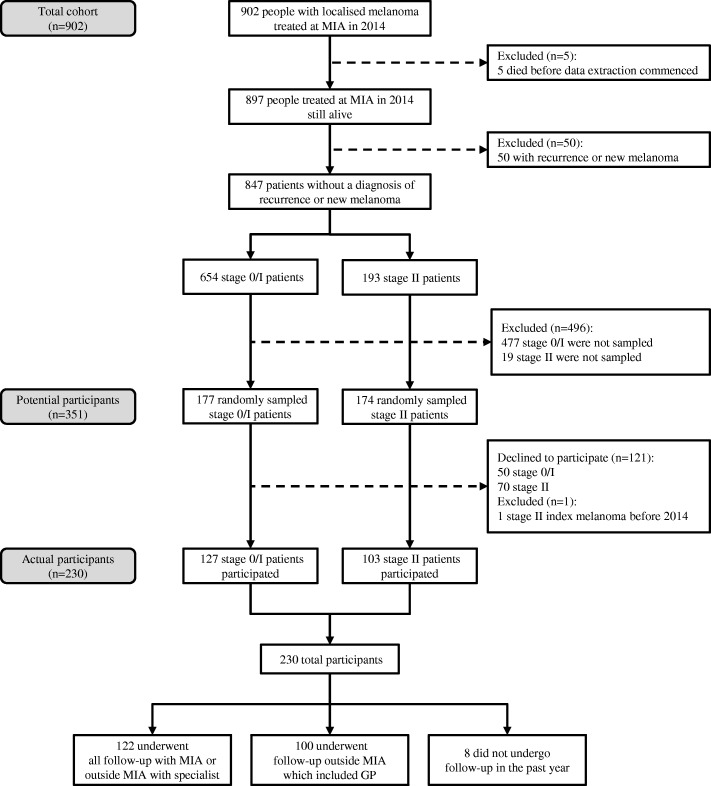
Selection of study participants. *MIA* Melanoma Institute Australia, *GP* general practitioner

We conducted a telephone interview among a stratified random sample of all patients in an inception cohort who were diagnosed with a first primary localised melanoma and had their treatment at MIA during the period between January 1st and December 31st 2014. MIA is a non-profit tertiary referral centre which specialises in melanoma research treatment and education [14]. Details of how potential participants were selected are provided in separate reports on a self-administered questionnaire on fear of melanoma recurrence [15] and phone interview questions on preferences for scheduled follow-up frequency [16]. Briefly, we randomly selected 351 people without recurrent or additional new primary melanoma, from a total of 897 people diagnosed and treated for localised melanoma in 2014 who were alive at the time of data collection. We planned a priori that we would report results for the group overall and separately by AJCC substage. We therefore used a stratified random sampling framework to ensure that there were sufficient numbers of people who had stage 0 to II melanoma. In order to achieve this, we randomly selected 177 patients with stage 0/I melanoma and 174 with stage II melanoma, giving a total of 351 potential participants. The flow of study participants is shown in Fig. 1.

### Telephone interview

We engaged an independent research organisation (Hunter Research Foundation) to conduct the telephone interviews. The interview questions were based on a survey questionnaire developed by the investigators, and included 15 questions relevant to the current study about shared care and routine test use (see Additional file 1, for the 15-item survey questionnaire). Participants were asked about visits they had undertaken to doctors in the past year that were specifically related to their melanoma diagnosis, and whether any tests were done for melanoma. Trained interviewers used Computer Assisted Telephone Interview (CATI) to administer the questions, with repeat calls to non-respondents in order to maximise the response rate. Interviews were conducted between September and November 2015, which was up to just under two years after their first primary melanoma diagnosis (range of time since diagnosis: 0.8 to 1.7 years). We asked about follow-up in the last year that was specifically related to melanoma, and occurred after their first primary melanoma diagnosis. Visits in the past year related to melanoma could thus refer to all visits from time of diagnosis, which might be less than one year in those with most recent date of diagnosis (e.g. 0 to 0.8 years post-diagnosis), or to visits in the year starting from a few months after diagnosis in those with an earlier date of diagnosis (e.g. 0.7 to 1.7 years post-diagnosis).

### Statistical analysis

We examined the following variables for participants who underwent shared care and those who did not: AJCC stage at initial presentation, anatomic site of primary lesion, time since diagnosis, history of non-melanoma skin cancer (NMSC), whether they had other chronic health problems, the number of different doctors seen last year for skin checks, education level, whether they lived with others, age at diagnosis, gender, remoteness of residence (based on postcode), and socio-economic status (based on postcode).

Continuous data were summarised as means with standard deviations if normally distributed, or medians and interquartile ranges if otherwise. Categorical data were presented as frequencies with percentages with 95% confidence intervals (CIs). For our main analysis, we undertook univariate analysis to compare characteristics for: [all follow-up at MIA or non-GP follow-up outside MIA] versus [any GP follow-up outside MIA]. As a sensitivity analysis, we also compared: [all follow-up at MIA] versus [any follow-up outside MIA, whether or not this included a GP]. We tested for statistical significance using the t-test for normally distributed continuous variables, the Chi-squared test for categorical variables, and the trend test for ordinal variables. We adjusted observed proportions and means to account for the oversampling of participants with index melanoma that was stage II because of the stratified sampling of our study design. The adjusted estimates are more representative of the full cohort of people treated at MIA without known recurrent or new primary melanoma, but do not account for differences between participants and non-participants.

All analyses were performed using Stata/IC 11.2 (StataCorp. 2009. Stata Statistical Software: Release 11. College Station, TX: StataCorp LP). We used svy, the survey prefix command to adjust estimated means and proportions for our sampling frame.

### Ethical and governance approval

This study was approved by the University of Sydney Human Research Ethics Committee and by the MIA Research Committee. All participants provided written consent to be in the study.

## Results

Of the 351 randomly sampled people who were invited to participate (potential participants), 230 consented to the telephone interview, giving a response rate of 66% (see Fig. 1). Phone interview participants (*n* = 230) had similar clinical and demographic characteristics to the potential participants (*n* = 351), but were more likely to have stage II disease than the full population of people treated for localised melanoma who did not have recurrent or additional new primary melanoma (*n* = 847) because of the stratified random sampling which oversampled for this.

Table 1 summarises the characteristics of the 122 individuals who either underwent all follow-up with MIA or only included specialists for any follow-up outside MIA, and the 100 individuals who chose to have some of their follow-up outside of MIA with a GP (an additional 8 individuals stated they had no follow-up in the past year). After adjusting for oversampling of stage II patients, we estimated that 61% (95% CI 54, 67%) of people with stage 0-II melanoma treated at MIA (and who are not known to have recurrent or new melanoma at up to 1.7 years post-diagnosis), choose shared care outside MIA with a GP. Compared with individuals who have all their follow-up at MIA or have follow up outside MIA which includes only specialists, individuals who have follow-up outside of MIA which includes a GP are more likely to be male (53% vs. 70%, *p* = 0.006), have lower educational level (15% vs. 38% have not finished secondary school, *p* < 0.001), live in more remote areas (83% vs. 66% live in major cities, *p* for trend< 0.001), and have a melanoma of lower AJCC stage (40% vs. 53% AJCC stage 0/IA, *p* for trend = 0.008). Not surprisingly, they also see a higher number of doctors for their skin follow-up (10% of people not in shared care with a GP vs. 31% of people in shared care with a GP have seen 3 different doctors in the last year, *p* for trend< 0.001). Comparing patients who have all follow-up at MIA to those who have any follow-up outside MIA (whether or not this includes a GP) gives similar results (see Additional File 2, for the characteristics of participants by type of follow-up care).

**Table 1:** Characteristics of people treated for localised melanoma by follow-up practice patterns^a^

	All follow-up with MIA or included follow-up with specialist outside MIA (*n* = 122)	Follow-up outside MIA which included local GP (*n* = 100)	Comparison of follow-up which did and did not include local GP (*p*-value)^b^	Total (*n* = 230)^c^
Age in years, mean (SD)	62.9 (14.3)	62.6 (11.9)	0.81	62.6 (13.2)
Gender			0.006	
Female	47 (39, 56)	30 (22, 39)		38 (33, 44)
Male	53 (45, 61)	70 (61, 78)		62 (56, 67)
Living with others	77 (69, 84)	77 (68, 84)	0.95	78 (72, 83)
Highest educational level			< 0.001	
Did not complete secondary school	15 (10, 22)	38 (29, 47)		26 (21, 32)
Completed secondary school	31 (24, 39)	14 (9, 21)		24 (19, 29)
Completed certificate or trade	27 (21, 35)	28 (20, 37)		27 (22, 32)
Completed university degree	27 (20, 35)	21 (14, 29)		24 (19, 29)
*Missing*				*(n = 1)*
SEIFA category^d^			0.34	
Low socio-economic status (deciles 1–3)	15 (10, 22)	20 (13, 28)		18 (13, 23)
Medium to High socio-economic status (deciles 4–10)	85 (78, 90)	80 (72, 87)		83 (77, 87)
Remoteness area^e^			< 0.001*	
Major cities of Australia	83 (76, 88)	66 (57, 75)		75 (69, 80)
Inner regional Australia	15 (10, 22)	26 (19, 34)		19 (15, 25)
Outer regional Australia	2 (1, 3)	8 (4, 15)		6 (4, 9)
Age at diagnosis in years, mean (SD)	60.9 (14.4)	60.8 (11.9)	0.85	60.7 (13.3)
More than a year since diagnosis	90 (84, 94)	92 (85, 96)	0.55	91 (87, 94)
AJCC substage			0.02*	
Stage 0	20 (14, 28)	25 (18, 35)		23 (18, 29)
Stage IA	20 (14, 28)	28 (20, 38)		25 (20, 31)
Stage IB	38 (30, 47)	30 (22, 39)		33 (27, 39)
Stage IIA	11 (9, 13)	10 (8, 12)		11 (10, 12)
Stage IIB/C	11 (9, 13)	7 (6, 9)		9 (8, 10)
Primary site of melanoma			0.15	
Limb	51 (42, 59)	38 (30, 48)		46 (40, 52)
Trunk	30 (23, 38)	37 (29, 46)		33 (27, 39)
Head or neck	20 (14, 27)	24 (17, 33)		22 (17, 27)
History of non-melanoma skin cancer (NMSC)	46 (38, 54)	59 (49, 67)	0.08	50 (44, 56)
Other chronic health problem	21 (15, 28)	26 (19, 35)	0.35	23 (18, 28)
No. of different doctors seen last year for skin checks			< 0.001*	
0	0	0		4 (2, 7)
1	56 (47, 64)	28 (21, 38)		41 (36, 47)
2	35 (27, 43)	41 (33, 50)		36 (31, 42)
3	10 (6, 15)	31 (23, 40)		19 (15, 24)

*AJCC* American Joint Committee on Cancer, *GP* general practitioner, *MIA* Melanoma Institute Australia, *SD* standard deviation, *SEIFA* Socio-Economic Indexes For Areas

*Trend test

^a^All values reported are column percentages (95% confidence intervals) unless otherwise indicated. Percentages were adjusted for stratified sampling from the total inception cohort. Some column totals do not add to 100% due to rounding

^b^*p*-values for comparison of [all follow-up with MIA + follow-up outside MIA not including GP] group versus follow-up outside MIA including GP group

^c^Included people who had no follow-up in the past year (*n* = 8)

^d^Based on Postal Area Index of Relative Socio-Economic Advantage and Disadvantage, Australian Bureau of Statistics 2011 [32]

^e^Based on 1270055006C190 Postcode 2012 to Remoteness Area 2011, Australian Bureau of Statistics 2011 [33]

Table 2 presents the frequency of shared care for the 222 people who attended at least one melanoma or other skin cancer related visit (scheduled or non-scheduled) either at MIA or elsewhere in the last year. Of these people, 132 had only scheduled visits, 79 had both scheduled and non-scheduled visits, and 11 people had only non-scheduled visits (in total 211 people attended at least one routinely scheduled visit and 90 people attended at least one non-scheduled visit; these categories are not mutually exclusive). After adjusting for oversampling of stage II patients, we estimate that among people attending scheduled visits, this includes a GP in 45% of individuals (95% CI 39, 52%), only a specialist outside of MIA in 31% (95% CI 25, 37%), and all scheduled visits at MIA in 24% (95% CI 19, 30%). Similarly, we estimate that among people attending non-scheduled visits, this includes a GP in 70% of individuals (95% CI 61, 78%), only a specialist outside of MIA in 27% (95% CI 20, 37%), and all non-scheduled visits at MIA in 3% (95% CI 1, 8%).

**Table 2:** Proportion of 222 people attending at least 1 follow-up visit in past year^a^

	All follow-up with MIA (*n* = 57)	Included follow-up outside MIA (*n* = 165)	
		Specialists only	Included GP
Scheduled skin follow-up	24 (19, 30)	31 (25, 37)	45 (39, 52)
Non-scheduled skin follow-up	3 (1, 8)	27 (20, 37)	70 (61, 78)

*GP* general practitioner, *MIA* Melanoma Institute Australia

^a^All values reported are row percentages (95% confidence intervals). Percentages were adjusted for stratified sampling from the total inception cohort. Excluded people who did not attend any follow-up in the past year (*n* = 8)

Experiences with care provided by health practitioners who were not doctors that were specifically related to their melanoma diagnosis, and whether clinicians (of all types) shared information is described in Table 3. Only 9% of people sought care related to their melanoma diagnosis from a non-doctor health practitioner, most commonly a nurse. When asked about whether the different doctors they had seen shared information with each other, 16% revealed that information was not shared, but only a few perceived problems with this.

**Table 3:** Use of non-doctor health practitioners for care of melanoma and clinician information sharing^a^

	All follow-up with MIA (*n* = 57)	Included follow-up outside MIA (*n* = 165)	No follow-up (*n* = 8)	Total (*n* = 230)
Non-medical care providers	5 (2, 13)	11 (7, 15)	17 (3, 56)	9 (7, 13)
Nurse^b^	4 (1, 13)	9 (5, 13)	17 (3, 56)	8 (5, 12)
Psychologist^b^	0 (0)	0.3 (0.1, 0.5)	0	0.2 (0.1, 0.4)
Physiotherapist^b^	1 (0.4, 2)	0	0	0.2 (0.1, 0.4)
Complementary medicine practitioner^b^	0 (0)	2 (1, 5)	0	1 (0.4, 4)
Different clinicians did not share information^c^	19 (11, 31)	16 (11, 22)	NA	16 (12, 22)
Problems because of this^b^	0	0.3 (0.1, 0.5)	NA	0.2 (0.1, 0.4)

*MIA* Melanoma Institute Australia, *NA* not applicable

^a^All values reported are column percentages (95% confidence intervals). Percentages were adjusted for stratified sampling from the total inception cohort

^b^The denominator for calculation of these percentages is the column total

^c^Missing data for people who did not have any follow-up visits in the past year (*n* = 8)

More than one third of all participants stated that they had undergone tests for melanoma in the past year (37% of participants, Table 4). The frequency of tests for melanoma was not statistically different between patients whose follow-up was at MIA or with a specialist outside MIA and patients whose follow-up was outside MIA which included a local GP. However, tests for melanoma were more likely to be performed during scheduled skin follow-up; in patients who were less than one year post-diagnosis; in patients with melanoma of a higher AJCC substage, and in patients who saw two or more doctors for skin checks (see Additional File 3, for factors associated with tests for melanoma). The number of people within each AJCC substage is small, but appears to show proportionately more testing in higher AJCC substages (70% of stage IIB/C, 53% of Stage IIA, 56% of Stage IB, 13% of stage IA, and 17% of stage 0, had a test). For the group overall, 13% had a blood test, 11% a chest X-ray, 10% an ultrasound, 8% a computed tomography (CT) scan, 6% a biopsy, 5% a positron emission tomography (PET) scan, and 2% a magnetic resonance imaging (MRI) scan (tests not mutually exclusive, and some participants had more than one; percentages adjusted for over-sampling of stage II patients).

**Table 4:** Tests for melanoma in the last year^a^

	Stage 0 (*n* = 36)	Stage IA (*n* = 39)	Stage IB (*n* = 52)	Stage IIA (*n* = 55)	Stage IIB/C (*n* = 48)^b^	Total (*n* = 230)^b,c^
Underwent any tests in the past year for surveillance of melanoma	6 (17)	5 (13)	29 (56)	29 (53)	32 (70)	101 (37)
Type of test performed^d^						
Chest X-ray	0 (0)	1 (3)	10 (19)	11 (20)	9 (20)	31 (11)
Blood test	2 (6)	3 (8)	8 (15)	13 (24)	13 (28)	39 (13)
CT scan	0 (0)	0 (0)	6 (12)	10 (18)	14 (30)	30 (8)
Biopsy	2 (6)	2 (5)	4 (8)	3 (6)	0 (0)	11 (6)
MRI	1 (3)	0 (0)	1 (2)	1 (2)	1 (2)	4 (2)
PET scan	1 (3)	0 (0)	3 (6)	4 (7)	7 (15)	15 (5)
Ultrasonography	0 (0)	1 (3)	9 (17)	9 (16)	9 (20)	28 (10)
Other^e^	0 (0)	0 (0)	1 (2)	1 (2)	0 (0)	3 (2)

*CT* computed tomography, *MRI* magnetic resonance imaging, *PET* positron emission tomography

^a^All values reported are frequencies (column percentages)

^b^Data are missing for 2 people who are not sure whether they had any tests for melanoma in the past year

^c^Percentages were adjusted for stratified sampling from the total inception cohort

^d^Some patients underwent more than one test in the past year

As reported previously, of the 262 participants interviewed in total, at the time of their interview, 13 had a recurrence (5%) and 19 had a new primary melanoma (7%) [16] (the 30 people with a recurrence and/or new primary melanoma are not included in the current report on shared care and routine testing). An additional 62 people reported they had a non-melanoma skin cancer detected during follow-up (24%), and 79 (30%) people had at least one of these events by the time of the interview (note that some people had more than one of these events). After adjusting for the over-sampling of people with stage II in our study, we estimate that in the full cohort, 20% (95% CI 16, 25%) have a recurrence, and/or new primary melanoma, and/or non-melanoma skin cancer. We were able to verify patient reports for recurrence and new primary melanoma in the MIA database, and found a high rate of people apparently unaware they had a recurrence or new primary melanoma [46% (*n* = 6/13) and 84% (*n* = 16/19) respectively]. The estimates for non-melanoma skin cancer are based on self-report only as these data are not recorded in the MIA database and true rates may be higher or lower than this. Half of those who stated that they were aware of their recurrence or new primary melanoma reported that this was first noticed (detected) by someone other than their specialist physician (by self for 3/7 people with a recurrence and 1/3 with a new primary, and by their GP in 1/7 people with a recurrence). Over two-thirds of those with a non-melanoma skin cancer during follow-up reported that this was first noticed (detected) by someone other than their specialist physician (by self for 21/58, relative or friend for 3/58, and GP for 16/58 people with non-melanoma skin cancer). All melanomas were treated by specialists (at MIA or outside MIA) whereas about half of non-melanoma skin cancers were treated by their GP (outside MIA).

## Discussion

Most people treated for localised melanoma at a large Australian melanoma specialist treatment centre, who have not had recurrent or additional new primary melanoma diagnosed, appear to choose shared care follow-up that includes a GP. Patients attend their GP for both scheduled and non-scheduled follow-up visits (45 and 70% of patients had at least one visit with a GP in the past year, for scheduled and non-scheduled visits respectively). The decision of whether to undergo shared care appears to be related to patient risk of a new primary or recurrent melanoma or new NMSC (AJCC stage and history of NMSC), gender, educational level, and remoteness of residence. More than a third of people have tests done for melanoma in the past year, including imaging such as chest X-rays, CT, MRI, and PET scans, much of which may be for melanoma surveillance. Detection and treatment of non-melanoma skin cancer is frequently done by the patient’s GP in the shared care setting outside of the specialist treatment centre.

An important strength of our study was that we utilised an epidemiological design for selecting potential participants from all individuals undergoing treatment for localised melanoma at a large specialist centre over a defined period of time (i.e. an inception cohort). We adjusted estimated means and proportions for the disproportionate stratified random sampling design so that our results would be representative of the full cohort. The telephone interview was administered centrally with the integration of CATI, which increases inter-interviewer reliability of the data collected and has been found to have good correlation with responses obtained through face-to-face interview [17]. Responses about new or recurrent melanoma were verified with a high quality database to ensure its reliability and accuracy.

There are also limitations to this study. The participants in our study had all undergone treatment at MIA, and our findings may not be generalisable to people treated for localised melanoma elsewhere. However, we may expect that the frequency of shared care would be at least this high outside of a specialist treatment centre. In addition, our findings may not provide an accurate picture of follow-up patterns in the full population treated at MIA for localised melanoma, given that 34% of participants approached declined to be interviewed (non-participants did not differ to actual participants in terms of baseline characteristics, including stage). As with any self-reported data, there is a potential for recall bias; self-report also meant that we missed some details on the data (e.g. whether an ultrasound was of lymph nodes or another anatomical site). We assumed that the 47% of people who did not provide a response to the question on whether any of the follow-up visits were for ‘other’ skin cancer, did not have a non-melanoma skin cancer. Likewise we assumed that the 10% who did not provide a response on how many visits were urgent or unscheduled, did not have any unscheduled visits. This may mean that we underestimated rates for both of these factors. We cannot be certain that the answers provided for the question on tests done for melanoma in the last year refer to surveillance on asymptomatic patients. The following observations suggest however, that many of the tests were done for surveillance purposes: there was a high proportion of imaging tests done; respondents were not have a known recurrence at the time of interview; tests were more likely to be done during scheduled skin follow-up, in patients who were less than one year post-diagnosis, and in patients with melanoma of a higher AJCC substage. Despite these limitations, our study provides a valid estimation of the volume of shared care practice and test use in patients with localised melanoma, of which limited data are currently available.

The high frequency of shared care found in this study may reflect the inconvenience of travel to the specialist centre cited in a previous study, with many preferring to alternate follow-up care with a local doctor closer to home [18]. Non-scheduled skin checks appeared to be especially likely to be done by local GPs. Other studies which included patients with higher risk of recurrence (AJCC stages III and IV) in Australia, [19] Germany, [20] and Netherlands [21] have also found that shared care with a GP is common. Evidence from a randomised controlled trial indicated that GP-led melanoma follow-up was feasible and improved patient satisfaction without adversely affecting psychosocial aspects of the disease [10]. Only a small number of participants in our study saw non-doctor health practitioners in addition to their doctors for follow-up. The high levels of fear of new or recurrent melanoma in this population [15] suggest that more people might benefit from seeing psychologists and nurses offering psychological support. Such support services appear to be available in rural, regional and urban areas in Australia, but may be currently under-utilised [22].

The apparent use of surveillance tests in our study is at odds with guidelines that recommend against routine imaging in this low risk group of people [13]. The number of tests undertaken increased with advancing melanoma substage, and the most common were: blood test, chest X-ray, CT scan, and regional lymph node ultrasound. This pattern was also observed in a German study, but with higher testing rates within all substages [20] which may reflect clinical guidance in that country [23]. Surveillance chest X-rays for the early detection of pulmonary metastases in stage I/II melanoma patients has been found to have low detection rates of recurrence (less than 10%) and no survival benefit [24–27]. The evidence on the usefulness of lymph node sonography is mixed, with some studies reporting lower detection rates than physical examination (including palpation) and others higher detection rates, [28–30] and conflicting results on survival benefit. In particular, the clinical utility of monitoring sentinel lymph nodes (if not removed at time of diagnosis) is not yet known. The uncertain benefits for routine surveillance tests must be balanced against the known costs of these tests, [31] which are especially substantial for imaging tests.

Further studies are required to prospectively evaluate the effectiveness and safety of shared care follow-up after localised melanoma. Randomised comparisons of clinically important outcomes such as time to detection and treatment of recurrence or new primary melanoma, and of NMSC, mortality, health-related quality of life, and cost-effectiveness are needed. Future research is also needed to determine the incremental value and clinical utility of routine surveillance tests above and beyond that of physical examination, for the early detection and treatment of recurrence or new primary melanoma, and appropriate time intervals for re-testing.

## Conclusions

The majority of people treated for localised melanoma at a specialist centre undergo shared care follow-up with a local GP. Many appear to be undergoing routine diagnostic imaging as part of their melanoma surveillance despite minimal evidence to support this practice.

## Additional files

Additional file 1: 15-item survey questionnaire. Survey questionnaire developed by the investigators, containing 15 questions about shared care and routine test use. (DOCX 15 kb)

Additional file 2: Characteristics of people treated for localised melanoma who had all follow-up at MIA compared with some follow-up outside MIA (with a specialist or GP). Same as file title. (DOCX 19 kb)

Additional file 3: Factors associated with routine investigations used during follow-up. Same as file title. (DOCX 17 kb)

## Abbreviations

AJCC: American Joint Cancer Committee
CATI: Computer Assisted Telephone Interview
CT: Computed tomography
GP: General practitioner
MIA: Melanoma Institute Australia
MRI: Magnetic resonance imaging
NMSC: Non-melanoma skin cancer
PET: Positron emission tomography
