# Supplementary material for: Use of shared care and routine tests in follow-up after treatment for localised cutaneous melanoma
Source: BMC Health Serv Res. 2018 Jun 20;18:477. doi: 10.1186/s12913-018-3291-7 (PMC6011416; doi:10.1186/s12913-018-3291-7)
Supplement: Supplementary file 1 — 15-item survey questionnaire. Survey questionnaire developed by the investigators, containing 15 questions about shared care and routine test use. (DOCX 15 kb) [file 12913_2018_3291_MOESM1_ESM.docx]

## Additional file 1: 15-item survey questionnaire

Q1. In the LAST YEAR, how many times have you seen ANY doctor for something to do with melanoma or other skin cancers, including routine follow-ups?

- Were any of these visits for routine melanoma follow-up?
- How many visits?
- Were any visits for melanoma urgent or non-routine visits?
- How many visits?
- What was the reason for the urgent or non-routine visit(s)?
- Did you make the appointment yourself?

Q2. Were any of these visits for ‘other’ skin cancer?

- How many visits?
- Were any of these visits urgent or non-scheduled?
- How many were urgent or unscheduled?
- Did you have any other visits to doctors for something to do with melanoma or other skin cancers, that we haven't covered?
- How many visits?
- What was the reason for this visit/these visits?

Q3. In the LAST YEAR did you miss any appointments for routine melanoma follow-ups?

- Can you tell me what stopped you from going to routine follow-up?

Q4. What is the name of a doctor you have seen in the last year for something to do with melanoma or other skin cancer?

- Why did you visit this doctor?
- Is this doctor a GP or a Specialist
- Where did you see this doctor?

Q5. Did you see another doctor for melanoma or other skin cancers in the LAST YEAR?

- What is the name of another doctor you have seen in the last year for melanoma or other skin cancer follow-up?
- Why did you visit this doctor?
- Is this doctor a GP or a Specialist?
- Where did you see this doctor?

Q6. Did you see another doctor for melanoma or other skin cancers in the LAST YEAR?

- What is the name of another doctor you have seen in the last year for melanoma or other skin cancer follow-up?
- Why did you visit this doctor?
- Is this doctor a GP or a Specialist
- Where did you see this doctor?

Q7. In the last year, have you seen anyone else besides a doctor for care of your melanoma, such as a nurse, psychologist, complementary medicine specialist or someone else?

- Who did you see?

Q8. In the last year, have the doctors you have seen had information from other doctors involved in your care; for instance, letters, test results or treatment plans?

- Were there any problems when this information wasn’t shared?

Q9. Have you had any tests for melanoma in the last year, such as chest x-ray, blood test, ‘CAT scan’, genetic test, or other sort of test?

Q10. What is the highest level of education you’ve had?

Q11. What is your current occupation; or previous occupation if not currently working?

Q12. What is your marital status?

Q13. Do you live alone or with others?

Q14. Do you have private health insurance?

Q15. What is your residential postcode?
