# Supplementary material for: Use of shared care and routine tests in follow-up after treatment for localised cutaneous melanoma
Source: BMC Health Serv Res. 2018 Jun 20;18:477. doi: 10.1186/s12913-018-3291-7 (PMC6011416; doi:10.1186/s12913-018-3291-7)
Supplement: Supplementary file 2 — Characteristics of people treated for localised melanoma who had all follow-up at MIA compared with some follow-up outside MIA (with a specialist or GP). Same as file title. (DOCX 19 kb) [file 12913_2018_3291_MOESM2_ESM.docx]

**Additional file 2: Characteristics of people treated for localised melanoma who had all follow-up at MIA compared with some follow-up outside MIA (with a specialist or GP)^a^**

|  | All follow-up with MIA  (n=57) | Included follow-up outside MIA  (n=165) | | | Comparison of follow-up with MIA and some outside MIA^b^ | No follow-up  (n=8) | Total  (n=230)^c^ |
| --- | --- | --- | --- | --- | --- | --- | --- |
|  |  | ***Specialists only***  ***(n=65)*** | ***Included GP***  ***(n=100)*** | ***Specialists only or included GP***  ***(n=165)*** |  |  |  |
| Age in years, mean (SD) | 62.9 (14.2) | 62.9 (14.4) | 62.6 (11.9) | 62.7 (13.0) | 0.88 | 59.4 (4.5) | 62.6 (13.2) |
| Gender |  |  |  |  | 0.003 |  |  |
| Female | 56 (43, 67) | 41 (30, 52) | 30 (22, 39) | 34 (28, 41) |  | 22 (6, 56) | 38 (33, 44) |
| Male | 45 (33, 57) | 59 (48, 70) | 70 (61, 78) | 66 (59, 72) |  | 78 (44, 94) | 62 (56, 67) |
| Living with others | 75 (63, 85) | 79 (68, 87) | 77 (68, 84) | 78 (71, 83) | 0.72 | 95 (88, 98) | 78 (72, 83) |
| Highest educational level |  |  |  |  | 0.007 |  |  |
| Did not complete secondary school | 19 (11, 30) | 13 (7, 23) | 38 (29, 47) | 28 (22, 35) |  | 36 (12, 70) | 26 (21, 32) |
| Completed secondary school | 37 (26, 50) | 26 (18, 37) | 14 (9, 21) | 19 (14, 25) |  | 41 (15, 73) | 24 (19, 29) |
| Completed certificate or trade | 30 (20, 42) | 25 (17, 36) | 28 (20, 37) | 27 (21, 34) |  | 5 (2, 13) | 27 (22, 32) |
| Completed university degree | 15 (8, 25) | 36 (26, 47) | 21 (14, 29) | 27 (21, 33) |  | 18 (3, 58) | 24 (19, 29) |
| *Missing* |  |  |  |  |  | *(n=1)* | *(n=1)* |
| SEIFA category^d^ |  |  |  |  | 0.32 |  |  |
| Low socio-economic status (deciles 1-3) | 22 (13, 34) | 10 (6, 18) | 20 (13, 28) | 16 (12, 22) |  | 22 (6, 56) | 18 (13, 23) |
| Medium to High socio-economic status (deciles 4-10) | 78 (66, 87) | 90 (82, 94) | 80 (72, 87) | 84 (78, 88) |  | 78 (44, 94) | 83 (77, 87) |
| Remoteness area^e^ |  |  |  |  | 0.003 |  |  |
| Major cities in Australia | 88 (80, 93) | 80 (69, 86) | 66 (57, 74) | 71 (65, 77) |  | 66 (32, 89) | 75 (69, 80) |
| Inner regional Australia | 10 (5, 19) | 19 (12, 29) | 26 (19, 34) | 23 (18, 30) |  | 0 | 19 (15, 25) |
| Outer regional Australia | 2 (1, 3) | 2 (1, 3) | 8 (4, 15) | 6 (3, 10) |  | 34 (11, 68) | 6 (4, 9) |
| Age at diagnosis in years, mean (SD) | 60.9 (14.3) | 60.9 (14.4) | 60.8 (11.9) | 60.8 (13.0) | 0.85 | 57.6 (4.4) | 60.7 (13.3) |
| More than a year since diagnosis | 90 (79, 95) | 90 (82, 95) | 92 (85, 96) | 91 (87, 95) | 0.67 | 100 | 91 (87, 94) |
| AJCC substage |  |  |  |  | 0.003* |  |  |
| Stage 0 | 20 (11, 34) | 20 (12, 31) | 25 (18, 35) | 23 (17, 30) |  | 34 (11, 68) | 23 (18, 29) |
| Stage IA | 12 (5, 24) | 26 (17, 38) | 28 (20, 37) | 27 (21, 34) |  | 51 (22, 79) | 25 (20, 31) |
| Stage IB | 41 (29, 53) | 37 (26, 49) | 30 (22, 39) | 32 (26, 40) |  | 0 | 33 (27, 39) |
| Stage IIA | 11 (9, 15) | 11 (9, 14) | 10 (8, 12) | 11 (9, 12) |  | 5 (2, 12) | 11 (10, 12) |
| Stage IIB/C | 16 (13, 21) | 7 (5, 9) | 7 (6, 9) | 7 (6, 8) |  | 10 (5, 21) | 9 (8, 10) |
| Primary site of melanoma |  |  |  |  | 0.38 |  |  |
| Limb | 49 (37, 61) | 52 (41, 63) | 38 (30, 48) | 44 (37, 51) |  | 61 (30, 85) | 46 (40, 52) |
| Trunk | 26 (17, 38) | 33 (23, 44) | 37 (29, 46) | 35 (29, 42) |  | 22 (6, 56) | 33 (27, 39) |
| Head/Neck | 25 (16, 38) | 15 (9, 25) | 24 (17, 33) | 21 (18, 27) |  | 17 (3, 56) | 22 (17, 27) |
| History of non-melanoma skin cancer (NMSC) | 41 (29, 53) | 50 (39, 61) | 59 (49, 67) | 55 (48, 62) | 0.02 | 5 (2, 12) | 50 (44, 56) |
| Other chronic health problem | 26 (17, 38) | 17 (10, 27) | 26 (19, 35) | 22 (17, 29) | 0.54 | 5 (2, 12) | 23 (18, 29) |
| No. of different doctors seen last year for skin checks |  |  |  |  | <0.001* |  |  |
| 0 | 0 | 0 | 0 | 0 |  | 100 | 4 (2, 7) |
| 1 | 75 (63, 84) | 41 (31, 53) | 28 (21, 38) | 34 (27, 41) |  | 0 | 41 (36, 47) |
| 2 | 22 (13, 34) | 45 (34, 56) | 41 (33, 50) | 42 (36, 50) |  | 0 | 36 (31, 42) |
| 3 | 4 (2, 5) | 14 (8, 24) | 31 (23, 39) | 24 (19, 31) |  | 0 | 19 (15, 24) |

AJCC: American Joint Committee on Cancer, GP: general practitioner, MIA: Melanoma Institute Australia, SD: standard deviation, SEIFA: Socio-Economic Indexes For Areas

*Trend test.

^a^All values reported are column percentages (95% confidence intervals) unless otherwise indicated. Percentages were adjusted for stratified sampling from the total inception cohort.

^b^p-values for comparison of all follow-up with MIA group versus follow-up outside MIA including specialist only or included GP group.

^c^Included people who had no follow-up in the past year (n=8).

^d^Based on Postal Area Index of Relative Socio-Economic Advantage and Disadvantage, Australian Bureau of Statistics 2011.[32]

^e^Based on 1270055006C190 Postcode 2012 to Remoteness Area 2011, Australian Bureau of Statistics 2011.[33]
