# Supplementary material for: Use of shared care and routine tests in follow-up after treatment for localised cutaneous melanoma
Source: BMC Health Serv Res. 2018 Jun 20;18:477. doi: 10.1186/s12913-018-3291-7 (PMC6011416; doi:10.1186/s12913-018-3291-7)
Supplement: Supplementary file 3 — Factors associated with routine investigations used during follow-up. Same as file title. (DOCX 17 kb) [file 12913_2018_3291_MOESM3_ESM.docx]

## Additional file 3: Factors associated with routine investigations^a^ used during follow-up^b^

| Factors | Underwent test for melanoma in the past year  (n=101) | Did not undergo test for melanoma in the past year  (n=127) | Comparison of participants who did or did not undergo test for melanoma  (p-value) |
| --- | --- | --- | --- |
| Type of follow-up care |  |  | 0.12 |
| All follow-up with MIA or with specialist outside MIA | 59 (50, 68) | 49 (41, 57) |  |
| Follow-up outside MIA which included local GP | 41 (32, 50) | 51 (43, 59) |  |
| Type of follow-up care |  |  | 0.15 |
| All follow-up with MIA | 28 (20, 36) | 19 (13, 25) |  |
| Follow-up outside MIA (specialist or local GP) | 70 (62, 78) | 77 (70, 83) |  |
| No follow-up in the past year | 2 (0.6, 8) | 5 (2, 9) |  |
| Scheduled skin follow-up | 98 (91, 100) | 91 (85, 95) | 0.04 |
| Non-scheduled skin follow-up | 44 (35, 54) | 36 (28, 44) | 0.16 |
| Age in years, mean (SD) | 62.1 (13.0) | 62.8 (13.0) | 0.82 |
| Gender |  |  | 0.33 |
| Female | 35 (27, 44) | 41 (33, 49) |  |
| Male | 65 (56, 73) | 59 (51, 67) |  |
| Living with others | 81 (73, 87) | 76 (68, 82) | 0.29 |
| Highest educational level |  |  | 0.34* |
| Did not complete secondary school | 24 (17, 33) | 28 (21, 35) |  |
| Completed secondary school | 22 (15, 30) | 25 (18, 32) |  |
| Completed certificate or trade | 29 (21, 38) | 26 (19, 33) |  |
| Completed university degree | 26 (18, 34) | 22 (16, 30) |  |
| *Missing* | *(n=1)* |  |  |
| SEIFA category^d^ |  |  | 0.17 |
| Low socio-economic status (deciles 1-3) | 22 (15, 31) | 15 (10, 22) |  |
| Medium to High socio-economic status (deciles 4-10) | 78 (69, 85) | 85 (79, 90) |  |
| Remoteness area^e^ |  |  | 0.36* |
| Major cities of Australia | 73 (34, 80) | 76 (69, 82) |  |
| Inner regional Australia | 20 (14, 28) | 19 (14, 26) |  |
| Outer regional Australia | 8 (4, 14) | 5 (2, 9) |  |
| Age at diagnosis in years, mean (SD) | 60.2 (13.0) | 60.9 (13.0) | 0.83 |
| Time since diagnosis |  |  | 0.03 |
| > 1 year | 87 (79, 92) | 94 (89, 97) |  |
| ≤ 1 year | 13 (8, 21) | 6 (3, 11) |  |
| AJCC substage |  |  | <0.001* |
| Stage 0 | 10 (5, 19) | 30 (23, 38) |  |
| Stage IA | 9 (4, 17) | 34 (27, 43) |  |
| Stage IB | 50 (41, 59) | 23 (17, 31) |  |
| Stage IIA | 15 (12, 18) | 8 (7, 9) |  |
| Stage IIB/C | 17 (14, 20) | 4 (3, 5) |  |
| Primary site of melanoma |  |  | 0.80 |
| Limb | 48 (39, 57) | 44 (37, 52) |  |
| Trunk | 31 (24, 40) | 34 (27, 42) |  |
| Head or neck | 20 (14, 29) | 22 (16, 29) |  |
| History of non-melanoma skin cancer | 51 (42, 60) | 49 (41, 24) | 0.61 |
| Other chronic health problem | 22 (15, 31) | 23 (17, 30) | 0.94 |
| No. of different doctors seen last year for skin checks |  |  | 0.001* |
| 0 (no follow-up in the past year) | 2 (0.6, 8) | 5 (2, 9) |  |
| 1 | 30 (22, 39) | 48 (40, 56) |  |
| 2 | 42 (33, 51) | 33 (26, 41) |  |
| 3 | 26 (19, 35) | 15 (10, 21) |  |

AJCC: American Joint Committee on Cancer, GP: general practitioner, MIA: Melanoma Institute Australia, SD: standard deviation, SEIFA: Socio-Economic Indexes For Areas

*Trend test.

^a^Routine investigations include any test, e.g. imaging, blood test, ultrasonography, etc. (see footnote of Table 4)

^b^All values reported are column percentages (95% confidence intervals) unless otherwise indicated. Percentages were adjusted for stratified sampling from the total inception cohort.

^c^Data are missing for 2 participants who are not sure whether they had any tests for melanoma in the past year.

^d^Based on Postal Area Index of Relative Socio-Economic Advantage and Disadvantage, Australian Bureau of Statistics 2011.[32]

^e^Based on 1270055006C190 Postcode 2012 to Remoteness Area 2011, Australian Bureau of Statistics 2011.[33]
